# Immunoinformatic-guided designing and evaluating protein and mRNA-based vaccines against *Cryptococcus neoformans* for immunocompromised patients

**DOI:** 10.1186/s43141-023-00560-3

**Published:** 2023-10-26

**Authors:** Amir Elalouf, Amit Yaniv-Rosenfeld

**Affiliations:** Department of Management, Bar-Ilan University, Ramat Gan, 5290002 Israel

**Keywords:** In silico vaccine, Cryptococcus neoformans, Mannoprotein, Fungal vaccine

## Abstract

**Background:**

*Cryptococcus neoformans* is a fungal pathogen that can cause serious meningoencephalitis in individuals with compromised immune systems due to HIV/AIDS (human immunodeficiency virus/acquired immunodeficiency syndrome), liver cirrhosis, and transplantation. Mannoproteins (MPs), glycoproteins in the *C. neoformans* capsule, crucially impact virulence by mediating adhesion to lung cells and modulating immune response via cytokine induction and phagocytosis influence. Therefore, creating a vaccine that can generate targeted antibodies to fight infection and prevent fungal illnesses is essential.

**Results:**

This research aims to create a unique, stable, and safe vaccine through bioinformatics methodologies, aiming at epitopes of T and B cells found in the MP of *C. neoformans.* Based on toxicity, immunogenicity, and antigenicity, this research predicted novel T cells (GNPVGGNVT, NPVGGNVTT, QTSYARLLS, TSVGNGIAS, WVMPGDYTN, AAATGSSSSGSTGSG, GSTGSGSGSAAAGST, SGSTGSGSGSAAAGS, SSGSTGSGSGSAAAG, and SSSGSTGSGSGSAAA) and B cell (ANGSTSTFQQRYTGTYTNGDGSLGTWTQGETVTPQTAYSTPATSNCKTYTSVGNGIASLALSNAGSNSTAAATNSSSGGASAAATGSSSSGSTGSGSGSAAAGSTAAASSSGDSSSSTSAAMSNGI, HGATGLGNPVGGNVTT, TMGPTNPSEPTLGTAI, GNPVGGNVTTNATGSD, and NSTAAATNSSSGGASA) epitopes for a multiple-epitope vaccine and constructed a vaccine subunit with potential immunogenic properties. The present study used four linkers (AAY, GPGPG, KK, and EAAAK linkers) to connect the epitopes and adjuvant. After constructing the vaccine, it was confronted with receptor docking and simulation analysis. Subsequently, the vaccine was cloned into the vector of *Escherichia coli* pET-28a ( +) by ligation process for the expression using the SnapGene tool, which confirmed a significant immune response. To assess the constructed vaccine’s properties, multiple computational tools were employed. Based on the MP sequence, the tools evaluated the antigenicity, immunogenicity, cytokine-inducing capacity, allergenicity, toxicity, population coverage, and solubility.

**Conclusion:**

Eventually, the results revealed a promising multi-epitope vaccine as a potential candidate for addressing global *C. neoformans* infection, particularly in immunocompromised patients. Yet, additional in vitro and in vivo investigations are necessary to validate its safety and effectiveness.

**Supplementary Information:**

The online version contains supplementary material available at 10.1186/s43141-023-00560-3.

## Background

*Cryptococcus neoformans* is a fungal pathogen that can cause severe meningoencephalitis in immunocompromised individuals, for instance, people with HIV/AIDS (human immunodeficiency virus/acquired immunodeficiency syndrome), liver cirrhosis, and transplant recipients [1]. The CDC (Centers for Disease Control and Prevention) reported that healthy individuals are unlikely to contract *C. neoformans* infections. However, the pathogen is responsible for many cases of cryptococcal meningitis in individuals with HIV/AIDS. The CDC estimated approximately 152,000 cases of cryptococcal meningitis eventuate annually worldwide among individuals with HIV/AIDS, with nearly 112,000 resulting in death. Moreover, Cryptococcus is now the leading cause of meningitis in adults in sub-Saharan Africa [2]. Zhao et al. (2023) strengthened the claim by noting that while *C. neoformans* infections are rare in individuals with healthy immune systems; they can cause significant illness in those with HIV/AIDS [3].

Although *C. neoformans* is not typically considered a cytotoxic fungal pathogen, there is ample evidence to suggest that it can cause damage to host cells and tissues. Symptoms of infection with *C. neoformans* comprise headache, fatigue, fever, and muscle aches, and in severe cases, the infection can progress to meningitis, which can be fatal [4, 5]. *C. neoformans* typically propagate through the respiration of aerosolized basidiospores and disperse to the central nervous system (CNS), leading to meningoencephalitis [6, 7]. Within the lungs, alveolar macrophages typically phagocytose *C. neoformans* cells. The disease commonly spreads through contact with fungus associated with various bird species, particularly pigeon feces and bat guano. Infection may also spread through contact with an infected individual [8–10].

Treatment for *C. neoformans* infection typically involves prescription antifungal medication for a minimum of 6 months and possibly longer depending on the severity and location of the infection [11]. Asymptomatic infections or mild-to-moderate pulmonary infections are commonly treated with fluconazole. Serious lung infections in the CNS are initially treated with amphotericin B combined with flucytosine, followed by fluconazole treatment for at least ten additional weeks [11, 12]. Treatment of invasive *C. neoformans* disease typically involves flucytosine, amphotericin B, and different azoles. However, treatment failures may still occur due to direct antifungal drug resistance [13–15]. While antifungal drug resistance is uncommon among clinical isolates of *C. neoformans*, it has been reported. Additionally, using antifungal drugs in long-term suppressive regimens has raised concerns about drug resistance development [16, 17]. However, a survey conducted at a university hospital between 1987 and 1994 to assess the susceptibility patterns of clinical isolates of *C. neoformans* found no evidence of the emergence of resistance, thus alleviating these concerns [18].

No vaccines are currently available for fungal infections, but ongoing research aims to develop vaccines, immunotherapy, and new drugs [19, 20]. Vaccines can stimulate the immune response and produce antibodies against fungal antigens, protecting against infection [21–26]. Fungal vaccines face limitations due to the commensal nature of fungi, their ability to establish clinical latency, and the lack of common antigens expressed in multiple genera of fungi [20]. However, fungal vaccines can be classified into different types based on their composition: whole organism, subunit, and conjugate. Antifungal drugs usually target fungal-specific structures like the cell membrane and cell wall, which are necessary for the survival of the fungus but not human cells. The fungal cell wall contains mannoproteins (MPs), β-glucans, and chitin/chitosan, which are essential for growth and survival and are targeted by antifungal drugs and the immune system [20, 27–31].

According to Ghanegolmohammadi et al. (2021) [32], MPs are predominantly found in the outer part of the fungal cell wall. They are essential for shape, cell rigidity, ion exchange, metabolism, and interactions with host defense mechanisms [32]. MP mutants in *Saccharomyces cerevisiae* were studied via high-dimensional morphological phenotyping. Yet, it remains unclear whether fungi can survive without MPs. Meanwhile, MPs are assembled and modified; they play a significant role in fungal pathogens’ virulence and/or cell wall integrity. The fungal cell wall also contains β-(1,6)-glucan, β-(1,3)-glucan, and chitin [29, 33]. Interestingly, MPs are unique to fungi and not present in humans [34, 35]. MPs possess various advantageous characteristics, including high conservation, abundance, immunogenicity, low risk of resistance development, and enhanced recognition by the host immune system [36]. These attributes collectively underscore the significant potential of MPs as a compelling target for vaccine development.

This study aimed to design a hypoallergic, non-toxic, and safe vaccine against *C. neoformans* using artificial intelligence. The vaccine was constructed of multiple antigenic, non-allergenic, immunogenic, cytokine inducers, and non-toxic B and T cell epitopes from the MP of the *C. neoformans*. The ensuing vaccine was analyzed using bioinformatic tools to assess its interaction with immune inducer receptors for activating the immune system, which was further examined for its immunogenic properties in real-world scenarios.

## Methods

**Fig. 1:**
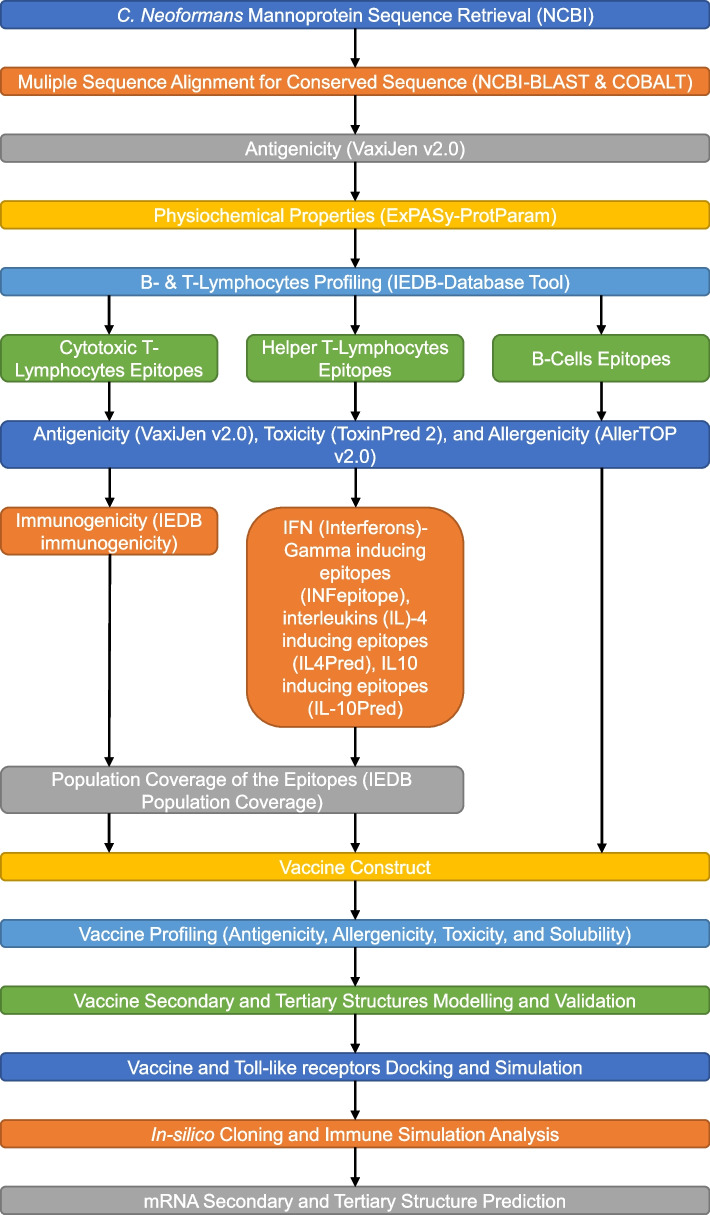
Outline for the in silico construction of the vaccine against *C. neoformans* and its subsequent validation

Figure 1 depicts the methodology employed for the in silico construction of the vaccine and its subsequent validation against *C. neoformans*.

### Protein sequence retrieval and multiple sequence alignment

The immunoreactive MP sequence of *C. neoformans* was obtained from NCBI with accession number XP_567104.1 and analyzed using NCBI-BLAST and COBALT to generate a multiple sequence alignment and identify conserved regions.

### Antigenic proteins

The antigenicity of the multiple sequence alignments of MP of *C. neoformans* was estimated using the VaxiJen v2.0 server [37–39].

### Physiochemical properties

The ExPASy-ProtParam online server [40, 41] evaluated the physiochemical properties of the selected sequence of the MP of *C. neoformans*. It calculates different physical and chemical parameters for protein sequences, including theoretical isoelectric point, molecular weight, extinction coefficients, grand average of hydropathicity, aliphatic index, instability index, positively and negatively charged residues, and estimated half-life.

### T cell and B cell epitope and feature profiling

The IEDB bioinformatics database tool [42] was used for T cell and B cell (LBL) epitope prediction, using different prediction methods such as Ab initio, homology-based, LBL epitope, T cell epitope, and structure-based prediction.

### CTL binding epitope prediction

The study used the CTL (cytotoxic T lymphocyte) binding epitopes prediction server to predict conserved CTL binding epitopes of the MP sequence of *C. neoformans* using the NetMHCpan EL 4.1 method [43]. The obtained epitopes were evaluated using VaxiJen v2.0, ToxinPred2 [44], immunogenicity [45], and AllerTOP v2.0 [46] servers to predict their antigenicity, toxicity, immunogenicity, and allergenicity, respectively.

### HTL binding epitope prediction

The conserved HTL (helper T lymphocyte) T cell binding epitopes of the MP of *C. neoformans* were predicted using the HTL binding prediction server with the IEDB recommended 2.22 method [42]. Each predicted epitope was evaluated for antigenicity, toxicity, IFN (Interferons)-Gamma inducing epitopes, interleukins (IL)-4 inducing epitopes, IL10 inducing epitopes, and allergenicity using various online servers, including VaxiJen v2.0, ToxinPred2, INFepitope [47], IL4Pred [48], IL-10Pred [49], and AllerTOP v2.0, respectively.

### LBL binding epitope prediction

The conserved LBL epitopes of the MP of *C. neoformans* were predicted using the antibody epitope prediction server using two methods: BepiPred linear epitope prediction 2.0 and Emini surface accessibility prediction [50] and an artificial neural network-based LBL epitope prediction server [51, 52]. VaxiJen v2.0, ToxinPred2, and AllerTOP v2.0 servers were used to predict the antigenicity, toxicity, and allergenicity of each predicted epitope.

### Population coverage of the epitopes

The population coverage calculation tool estimates the percentage of a population covered by a given set of epitopes based on their conservation across different individuals. It uses data from the IEDB’s database of experimentally determined epitopes and population genetic studies. The Israeli population was selected for calculating the population coverage of individual MHC class-I and class-II epitopes using IEDB’s population coverage [53].

### Epitope conservancy analysis

The conservancy of selected antigenic epitopes from the MP of *C. neoformans* was analyzed using the Epitope Conservancy Analysis tool [54].

### Vaccine construction

The constructed vaccine for *C. neoformans* consisted of linked antigenic epitopes of CTL, HTL, and LBL, as well as an adjuvant linked together by AAY, GPGPG, KK, and EAAAK linkers [55–57]. The vaccine sequence began with a 50S ribosomal protein L7/L12 adjuvant (UniProt ID: P0A7K2) and ended with a 6-His tag [58].

### Physiochemical parameters, antigenicity, allergenicity, toxicity, and solubility of vaccine construct

The physicochemical properties of the vaccine were evaluated using the ExPASy-ProtParam online server [40, 41] physical and chemical parameters. The vaccine’s antigenicity, allergenicity, and toxicity were evaluated using VaxiJen 2.0, AllerTop 2.0, and Toxinpred2 online servers, respectively. SoluProt [59] was used to predict the soluble protein expression in *E. coli*.

### Secondary and tertiary structures modeling

The secondary structure parameters of the *C. neoformans* vaccine construct were predicted using the SOPMA online server [60] with default settings, and the graphical representation was obtained. The tertiary structure was predicted using ColabFold [61], which uses AlphaFold2 and Alphafold2-multimer and generates sequence templates through HHsearch and MMseqs2.

### Refinement and verification of 3D vaccine

The 3D structure of the vaccine construct of *C. neoformans* was refined using GalaxyRefine [62] online web server, which repacks and rebuilds the side chains to relax the structure by molecular dynamics simulation. The refined structure was then validated using PROCHECK, ERRAT, and Verify3D. PROCHECK [63] analyzes residue-by-residue and overall structure geometry to build the Ramachandran Plot. ERRAT examines non-covalent interactions among diverse atom types and graphs the error function values against a sliding window of nine residues. Finally, verify3D categorizes residues into structural classes and compares the outcomes with established high-quality structures.

### Discontinuous and linear B cell epitope prediction

The IEDB server’s ElliPro tool [64] confirmed the presence of discontinuous and linear B cell epitopes in the vaccine.

### Molecular docking and simulation

The vaccine’s binding affinity with Toll-like receptors (TLR)-2, TLR4, and TLR6 was evaluated using ClusPro 2.0 [65–68]. The 3D structures of TLR2, TLR4, and TLR6 were obtained from the Protein Data Bank (PDB) and AlphaFold protein structure database. Ligands and heteroatoms were removed from the TLR proteins and uploaded to the ClusPro 2.0 server with the vaccine as a ligand for protein–protein docking. The resulting complexes were subjected to molecular dynamics simulation using the iMODS [69, 70] server to analyze the NMA for determining collective motion in internal coordinates and torsional angles of the vaccine-TLR complexes. Essential dynamics were utilized for protein stability and motion prediction based on various factors.

### Codon optimization and in silico cloning

In order to analyze the expression of the vaccine in *E. coli* K12, JCAT [71] was utilized to adapt codons for efficient ribosome binding, transcription termination, and restriction enzyme cleavage site. SnapGene 4.2 [72, 73] software was used for cloning, introducing SgrAI and HpaI restriction sites to the vaccine sequence and then inserting them into the *E. coli* pET28a( +) expression vector.

### mRNA secondary and tertiary structure prediction

We used the Transcription and Translation Tool [74] to predict the secondary and tertiary structures of the vaccine mRNA and convert the optimized DNA sequence to RNA sequence. The mRNA secondary structure was then predicted using the RNAfold web server [75–77] for thermodynamic analysis and minimal free energy score. Finally, we utilized the 3dRNA/DNA [78] web server to predict the 3D structure of the single-stranded mRNA.

### Immune simulation analysis

The online antigen-based immune simulator C-ImmSim [79, 80] was used to assess the immunogenic profile of the vaccine. The prediction of immune reactions by this web server is based on a hybrid approach that combines the position-specific scoring matrix (PSSM) with a machine learning algorithm. The vaccine was administered in three doses of 1000 antigens with an 8-week gap between doses. The doses were given at time-step 168, 504, and 1008 (representing 8 h in real life), respectively, with the first dose given at time-step 1. The simulation was run for 1050 time steps with default parameters. The resulting figures were interpreted using Simpson’s Diversity Index (D) [74, 81].

## Results

### Protein sequence retrieval and multiple sequence alignment

The conserved sequence for the vaccine development was chosen by performing a multiple sequence alignment of the MP sequence of *C. neoformans*.

### Antigenic proteins

The MP of *C. neoformans* was found to be antigenic with a score of 0.8760 at a threshold level of 0.4, as confirmed by VaxiJen v2.0.

### Physiochemical properties

The selected sequence of MP of *C. neoformans* was analyzed by ProtParam to determine its physiochemical properties (Table 1). The protein has a molecular weight of 38.157 kDa, 377 amino acids, and a theoretical pI of 4.03. It has an extinction coefficient of 54360 M-1 cm-1 at 280nm and a slightly hydrophilic GRAVY score of − 0.113. The protein has an instability index of 24.89 and an aliphatic index of 64.32 and is classified as stable. The protein has different estimated half-lives in different organisms: 20 h in mammalian reticulocytes in vitro, 30 min in yeast in vivo, and over 10 h in *E. coli *in vivo. The protein also contains 8 positively charged residues (Arg + Lys) and 31 negatively charged residues.

**Table 1:** Physiochemical properties of MP of *C. neoformans* predicted by ProtParam

Sr. No	Physiochemical properties	MP
**1**	Molecular weight (kDa)	38.157
**2**	Amino acids number	377
**3**	Theoretical pI	4.03
**4**	Ec (M^−1^ cm^−1^, at 280nm)	54360
**5**	GRAVY	− 0.113
**6**	II	24.89
**7**	AI	64.32
**8**	R^+^	8
**9**	R^−^	31
**10**	Protein classification	Stable
**11**	Estimated half-life	20 h (mammalian reticulocytes, in vitro), 30 min (yeast, in vivo), and > 10 h (*E. coli*, in vivo)

### T cell and B cell epitope prediction

An IDEB server was utilized to predict binding epitopes of MP of *C. neoformans* for CTL, HTL, and LBL.

### CTL binding epitope prediction

The IDEB server utilized NetMHCpan EL 4.1 to predict 9963 CTL binding epitopes of the *C. neoformans* MP sequence. Table 2 displays the filtered CTL binding epitopes selected based on their antigenicity, non-allergenicity, immunogenicity, and non-toxic properties. Table S1 from the supplementary material shows the alleles of the selected CTL epitopes for vaccine designing.

**Table 2:** NetMHCpan EL 4.1 method on IEDB server predicted antigenic CTL binding epitopes of MP in *C. neoformans*

Sr. No	Position	Peptides	Antigenicity Score	Toxicity	Immunogenicity	Allergenicity
**1**	141–149	GNPVGGNVT	4.03	No	0.12	No
**2**	142–150	NPVGGNVTT	3.45	No	0.13	No
**3**	45–53	QTSYARLLS	1.93	No	0.013	No
**4**	277–285	TSVGNGIAS	1.95	No	0.21	No
**5**	200–208	WVMPGDYTN	2.56	No	0.0052	No

### HTL binding epitope prediction

The IEDB recommended the 2.22 method, which predicted 9801 HTL binding epitopes of the MP of *C. neoformans*. The predicted epitopes were filtered based on their antigenicity, non-allergenicity, IFN-gamma inducing, IL4 inducing, IL10 inducing, and non-toxic properties, as shown in Table 3. Table S2 from supplementary material shows the alleles of the selected HTL epitopes for vaccine designing.

**Table 3:** MP of *C. neoformans* antigenic HTL binding epitopes predicted using IEDB recommended 2.22 method on the IEDB server

Sr. No	Position	Epitopes	Antigenicity Score	Toxicity	IFN-Gamma Inducing ability	IFN-Gamma Inducing Score	IL4 Inducing	IL10 Inducing	Allergenicity
**1**	309–323	AAATGSSSSGSTGSG	2.45	No	Positive	0.86	Positive	Positive	No
**2**	318–332	GSTGSGSGSAAAGST	2.79	No	Positive	0.85	Positive	Positive	No
**3**	317–331	SGSTGSGSGSAAAGS	2.86	No	Positive	0.99	Positive	Positive	No
**4**	316–330	SSGSTGSGSGSAAAG	2.91	No	Positive	0.84	Positive	Positive	No
**5**	315–329	SSSGSTGSGSGSAAA	2.91	No	Positive	1.14	Positive	Positive	No

### LBL binding epitope prediction

The Emini surface accessibility prediction, BepiPred linear epitope prediction 2.0, and Artificial neural network-based LBL epitope prediction methods were utilized to predict the LBL epitopes of MP of *C. neoformans*. The predicted LBL binding epitopes were screened for antigenicity, non-allergenicity, and non-toxic properties and are presented in Table 4.

**Table 4:** Predicted MP LBL epitopes of *C. neoformans* using IEDB’s Emini surface accessibility prediction, BepiPred Linear Epitope Prediction 2.0, and artificial neural network-based LBL epitope prediction methods

Sr. No	Position	Peptide	Length	Antigenicity Score	Toxicity	Allergenicity
**1**	228–353	ANGSTSTFQQRYTGTYTNGDGSLGTWTQGETVTPQTAYSTPATSNCKTYTSVGNGIASLALSNAGSNSTAAATNSSSGGASAAATGSSSSGSTGSGSGSAAAGSTAAASSSGDSSSSTSAAMSNGI	126	1.2711	No	No
**2**	135–151	HGATGLGNPVGGNVTT	16	3.1745	No	No
**3**	28–44	TMGPTNPSEPTLGTAI	16	1.0603	No	No
**4**	141–157	GNPVGGNVTTNATGSD	16	2.5732	No	No
**5**	294–310	NSTAAATNSSSGGASA	16	1.628	No	No

### Population coverage of the epitopes

**Fig. 2:**
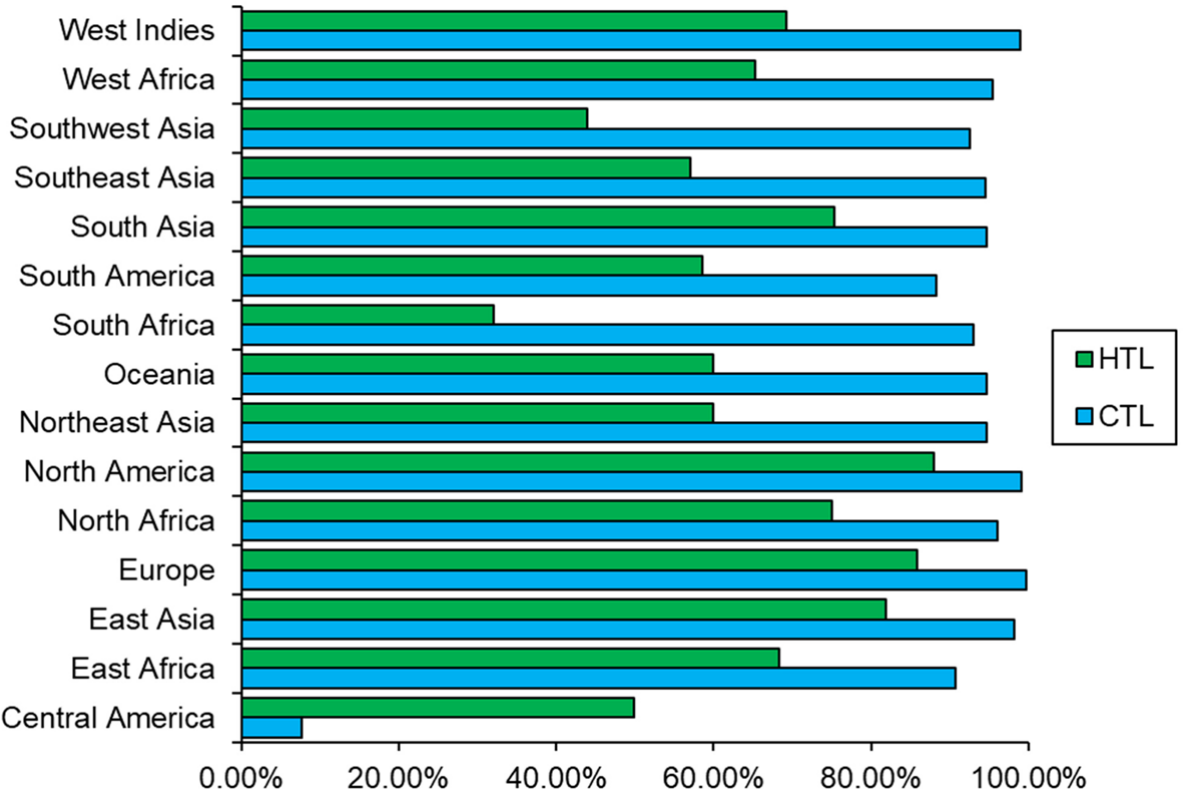
IEDB’s population coverage tool assesses the geographic distribution of population coverage percentages for CTL and HTL epitopes

IEDB’s Population Coverage was used to determine the population coverage percentages of CTL and HTL epitopes in different regions, as illustrated in Fig. 2.

### Epitope conservancy analysis

All selected CTL, HTL, and LBL epitopes for an MP of the *C. neoformans* vaccine were confirmed as conserved by epitope conservancy analysis.

### Protein-based vaccine construction

**Fig. 3:**
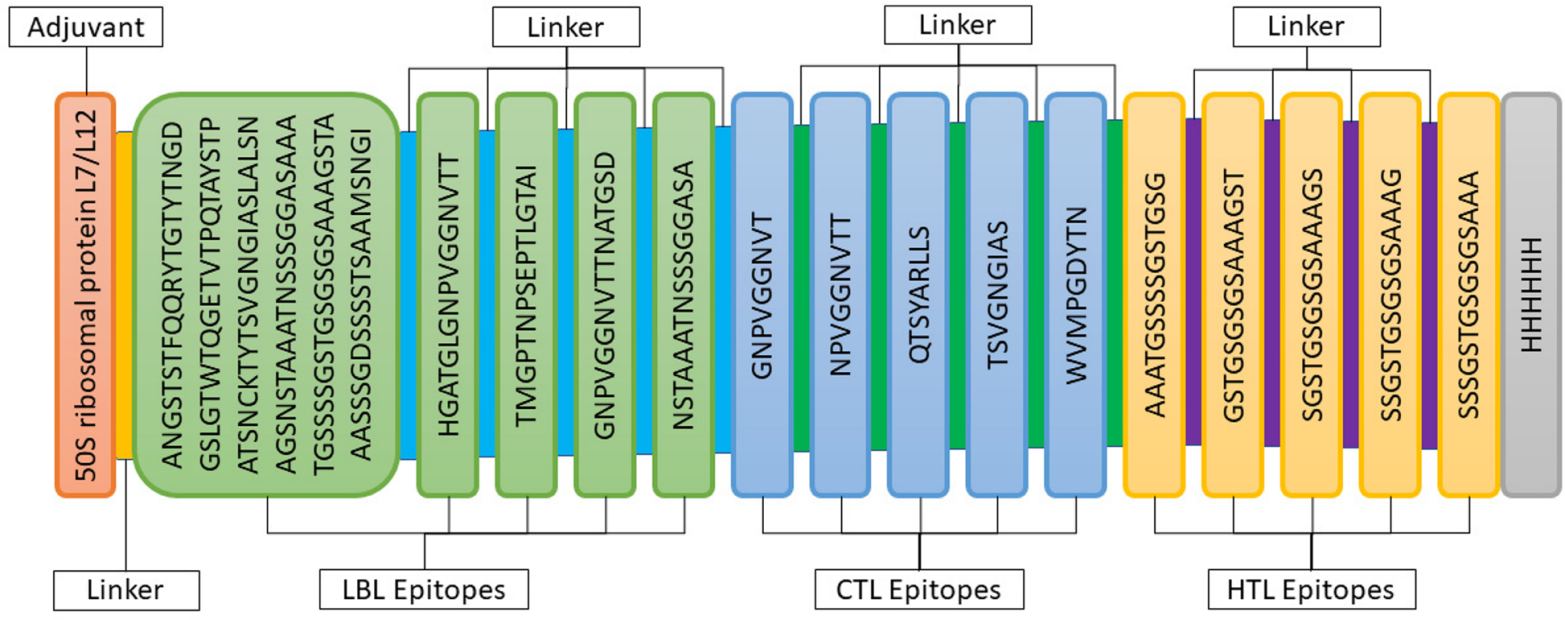
Structure of the *C. neoformans* vaccine candidate’s MP, including adjuvant, LBL, CTL, and HTL epitopes, separated by linkers and a 6-H tag

The vaccine for MP of *C. neoformans* contained 5 CTL, 5 HTL, and 5 LBL epitopes, an adjuvant at the N-terminal end combined with different linkers, and a 6 × His tag at the C-terminal end, as shown in Fig. 3.

### Physiochemical parameters, antigenicity, allergenicity, toxicity, and solubility of vaccine construct

The ProtParam server assessed the physiochemical properties of the MP vaccine of *C. neoformans* (Table 5). The VaxiJen 2.0 server verified its antigenicity with a score of 0.6466, and AllerTop 2.0 and Toxinpred2 confirmed the vaccine’s non-allergenic and non-toxic properties (Table 5). The constructed vaccine also has a high solubility score of 0.902, indicating its soluble expression in *E. coli* (Table 5).

**Table 5:** Properties of *C. neoformans* MP vaccine: physiochemical, antigenic, allergenic, toxic, and soluble

Sr. No	Physiochemical properties	Vaccine construct
**1**	Molecular weight	45,586.01
**2**	Number of amino acids	490
**3**	Theoretical pI	6.18
**4**	Formula	C_1903_H_3051_N_567_O_716_S_8_
**5**	Ec (M^−1^ cm^−1^, at 280nm)	27390
**6**	GRAVY	− 0.226
**7**	II	17.10
**8**	AI	54.22
**9**	R^+^	26
**10**	R^−^	29
**11**	Stability	Stable
**12**	Estimated half-life	30 h (mammalian reticulocytes, in vitro) > 20 h (yeast, in vivo) > 10 h (*E. coli*, in vivo)
**13**	Antigenicity score	1.4344
**14**	Antigenicity	Antigenic
**15**	Allergenicity	Non-allergenic
**16**	Toxicity	Non-toxic
**17**	Solubility score	0.88

### Secondary and tertiary structure modeling

**Fig. 4:**
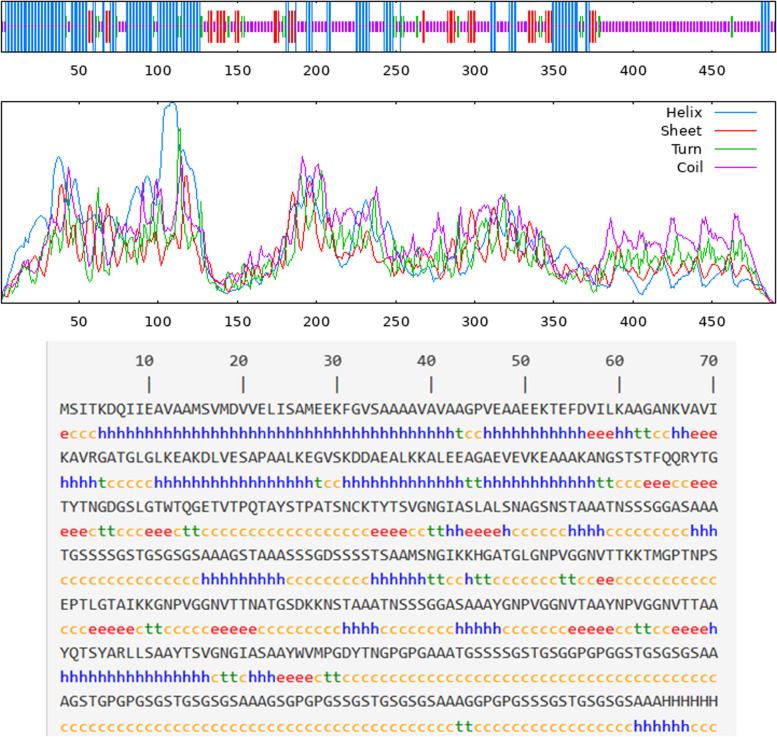
Graphical representation of the *C. neoformans* vaccine construct’s secondary structure

**Fig. 5:**
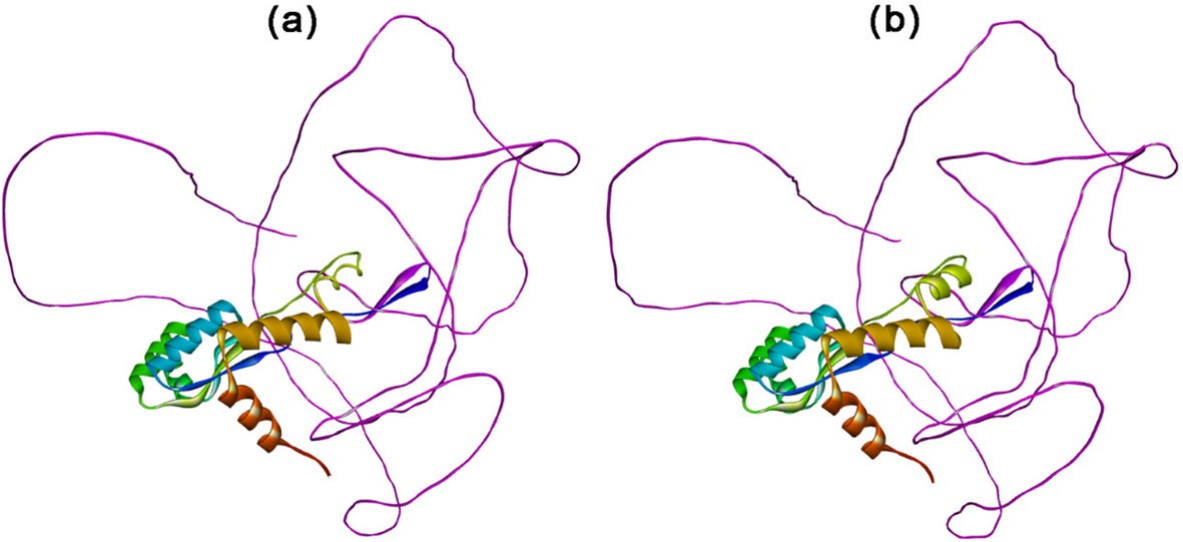
The 3D structure of *C. neoformans* MP vaccine construct predicted by ColabFold (**a**) and refined by GalaxyRefine (**b**)

SOPMA webserver predicted secondary structure parameters of the constructed vaccine for the MP of *C. neoformans*. Table 6 and Fig. 4 show the percentage of the vaccine’s alpha helix, extended strand, beta-turn, and random coil. The random coil was the dominant structure (50.41%). ColabFold generated five 3D models based on C-score, with Fig. 5a displaying the tertiary structure of the vaccine protein.

**Table 6:** Predicting secondary structure parameters of *C. neoformans* MP vaccine construct

Sr. No	Secondary structure parameters	Percentages
**1**	**Alpha helix (%)**	32.65
**2**	**Extended strand (%)**	10.61
**3**	**Beta turn (%)**	6.33
**4**	**Random coil (%)**	50.41

### Refinement and verification of 3D vaccine

**Fig. 6:**
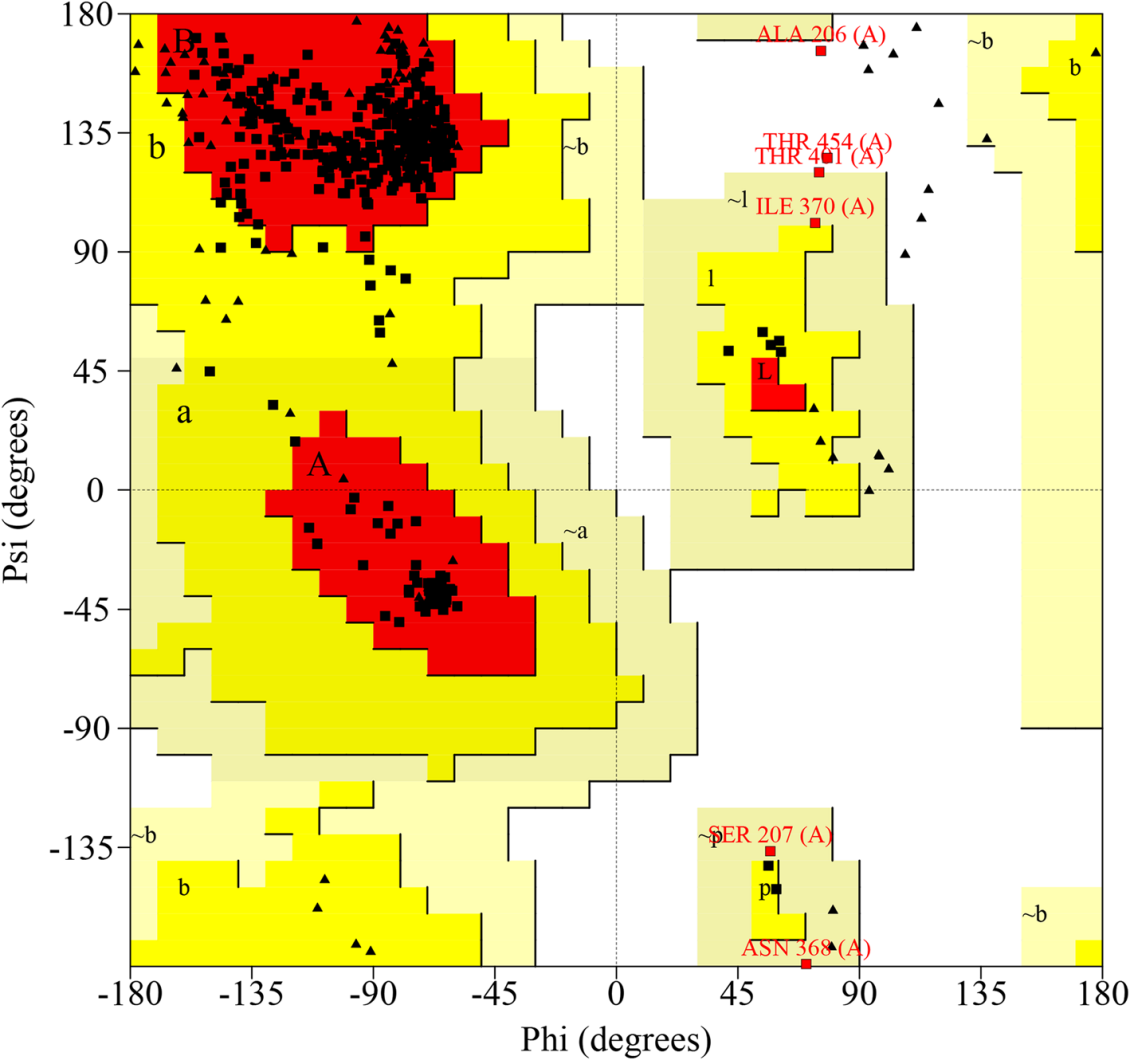
Ramachandran plot used to validate the 3D structure of *C. neoformans* MP vaccine construct

The 3D structure of the vaccine of MP of the *C. neoformans* was refined using GalaxyRefine online web server (Fig. 5b) and validated with a Ramachandran plot (Fig. 6). The plot showed more than 90% of the residues in the most favored region, indicating a good model. The number of residues in the disallowed region of the Ramachandran plot was only 0.8%. The ERRAT overall quality factor was 94.697%. However, the VERIFY3D averaged 3D-1D score failed with 73.27%, less than 80% of the amino acids scored.

### Discontinuous and linear B cell epitope prediction

**Fig. 7:**
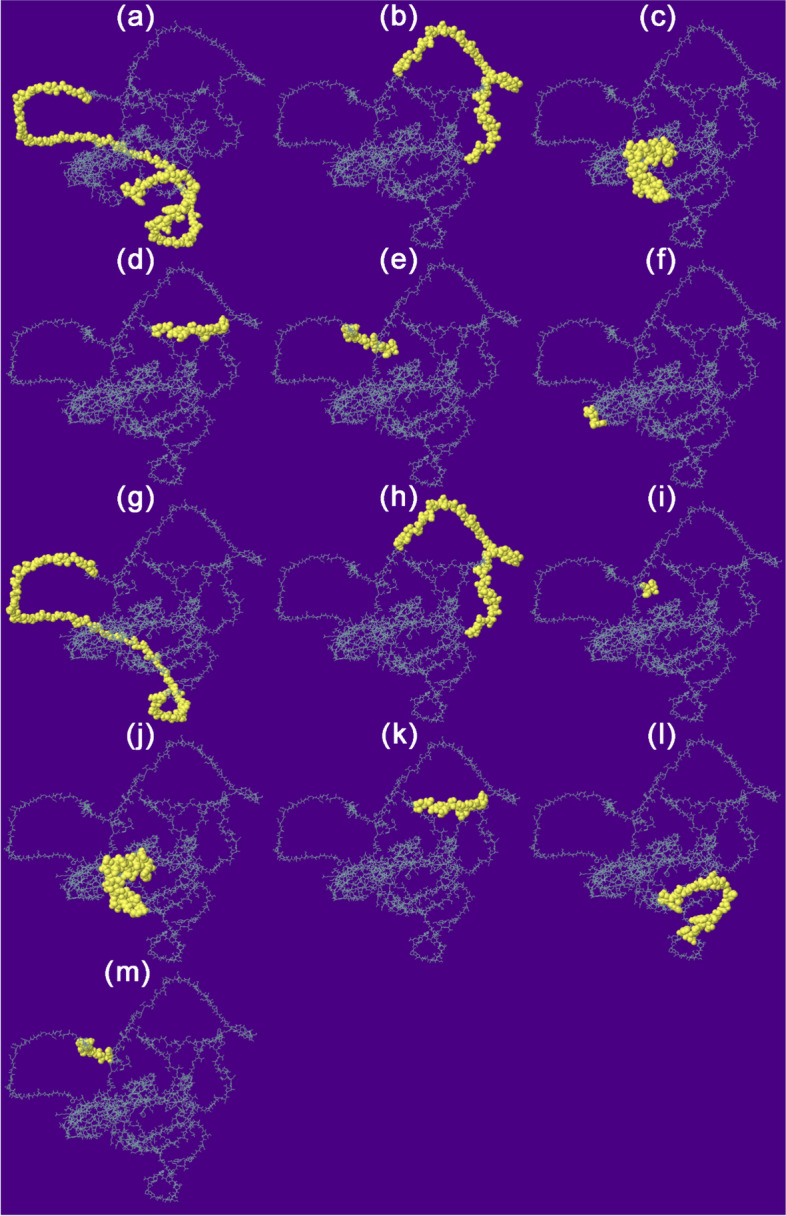
Linear and discontinuous B cell epitopes mapped on the vaccine construct of MP of *C. neoformans*. The yellow area of the vaccine shows each linear B cell epitope containing residues from 5 to 113 with score values from 0.505 to 0.756 (**a**–**e**) and each discontinuous B cell epitope containing residues from 3 to 86 with score values from 0.56 to 0.808 (**f**–**m**). Information on the number, types of residues, and scores of linear and discontinuous B cell epitopes can be found in Tables S3 and S4 from supplementary material, respectively

ElliPro tool confirmed the presence of five linear B cell epitopes and 14 discontinuous B cell epitopes (Fig. 7) in the vaccine of MP of the *C. neoformans*, with score values in Tables S3 and S4 from supplementary material. The presence of these epitopes is essential for activating humoral immunity and the secretion of antibodies against the foreign antigen.

### Molecular docking and simulation

**Fig. 8:**
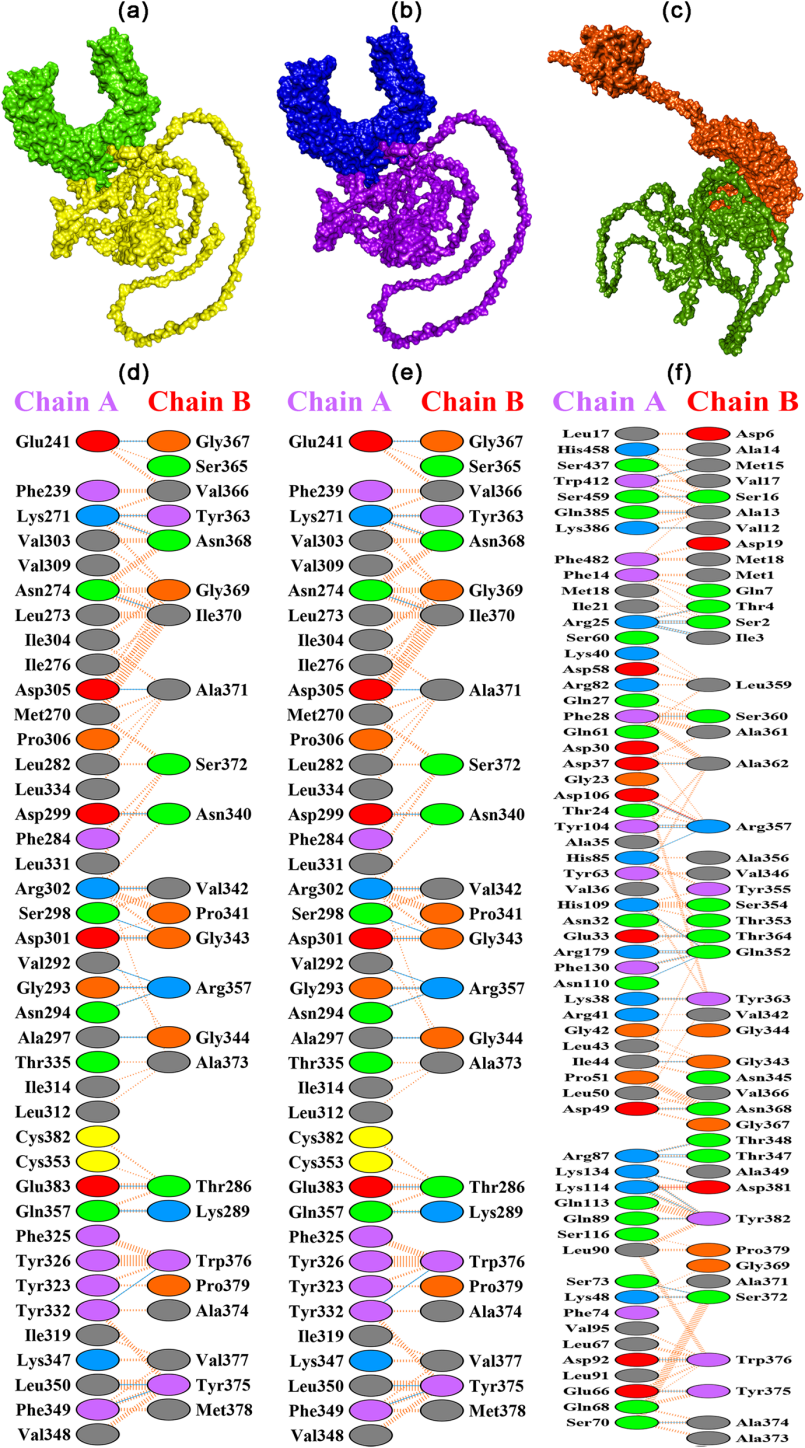
ClusPro 2.0 provided the best vaccine-TLR2, vaccine-TLR4, and vaccine-TLR6 docking results. The vaccine’s docking positions with TLR2, TLR4, and TLR6, and the interactions between the vaccine’s residues and TLR2, TLR4, and TLR6, are shown in **a**–**c** and **d**–**f**, respectively

ClusPro 2.0 was used to perform vaccine-TLR2, vaccine-TLR4, and vaccine-TLR6 docking and estimated the binding affinity of 30 different complexes. The best dock complexes of vaccine-TLR2, vaccine-TLR4, and vaccine-TLR6 were visualized in PyMol and Discovery Studio, with respective binding affinity − 1413.7, − 1413.7, and − 1390.2 kcal/mol, as shown in Fig. 8 and Table 7.

**Table 7:** The properties and characteristics of the best vaccine-TLR2, vaccine-TLR4, and vaccine-TLR6 docking complexes obtained from ClusPro 2.0, including the number of interface residues, salt bridges, hydrogen bonds, non-bonded contact with interface area, binding affinity, electrostatic-favored binding affinity, hydrophobic-favored binding affinity, and van-der wall and electrostatic binding

Docking	No. of interface residues	Interface Area (Å2)	Binding Affinity (kcal/mol)	Electrostatic-favored binding affinity (kcal/mol)	Hydrophobic-favored binding affinity (kcal/mol)	Van-der Waal and electrostatic binding affinity (kcal/mol)	No. of salt bridges	No. of hydrogen bonds	No. of non-bonded contacts
**TLR2-vaccine**	40–24	1466–1882	− 1413.7	− 1714.8	− 2238.2	− 346.5		18	264
**TLR4-vaccine**	40–24	1466–1882	− 1413.7	− 1714.8	− 2238.2	− 346.5		18	264
**TLR6-vaccine**	60–47	2340–2594	− 1390.2	− 1340.6	− 1972.2	− 209.1	2	36	370

**Fig. 9:**
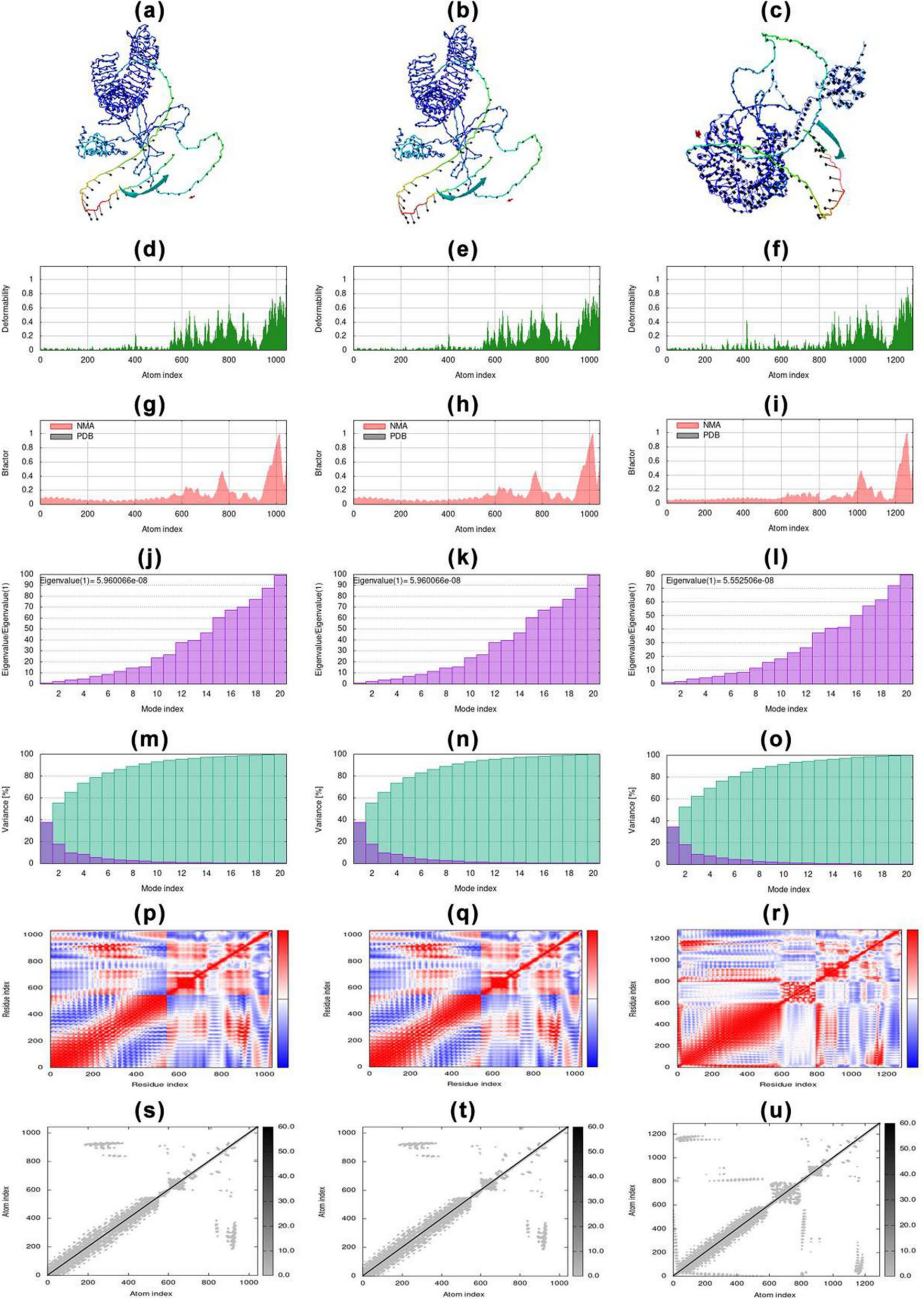
MDS results of vaccine-TLR2, vaccine-TLR4, and vaccine-TLR6 docked complexes obtained using iMODs server. The results include NMA mobility (**a**–**c**), deformability (**d**–**f**), B-factor (**g**–**i**), eigenvalues (**j**–**l**), percentage variance (**m**–**o**), covariance map (**p**–**r**), and elastic network map (**s**–**u**) of the complexes

The stability and mobility of the vaccine-TLR2, vaccine-TLR4, and vaccine-TLR6 docked complexes were analyzed using the iMODS tool based on dynamics and normal modes. The mobility of residues and docked complexes were shown with small and large arrows, respectively, and deformability values were shown in Fig. 9a–f. The B-factor values obtained from NMA indicated the mobility of docked complexes, and eigenvalues represented the rigidity of the complexes (Fig. 9g-l). The variance graphs displayed the relative contributions of each normal mode’s variance to the equilibrium motions. The covariance graphs showed the mobility types of a particular molecule region, and elastic network graphs displayed the stiffness of the springs that link pairs of atoms (Fig. 9m–u).

### Codon optimization and in silico cloning

**Fig. 10:**
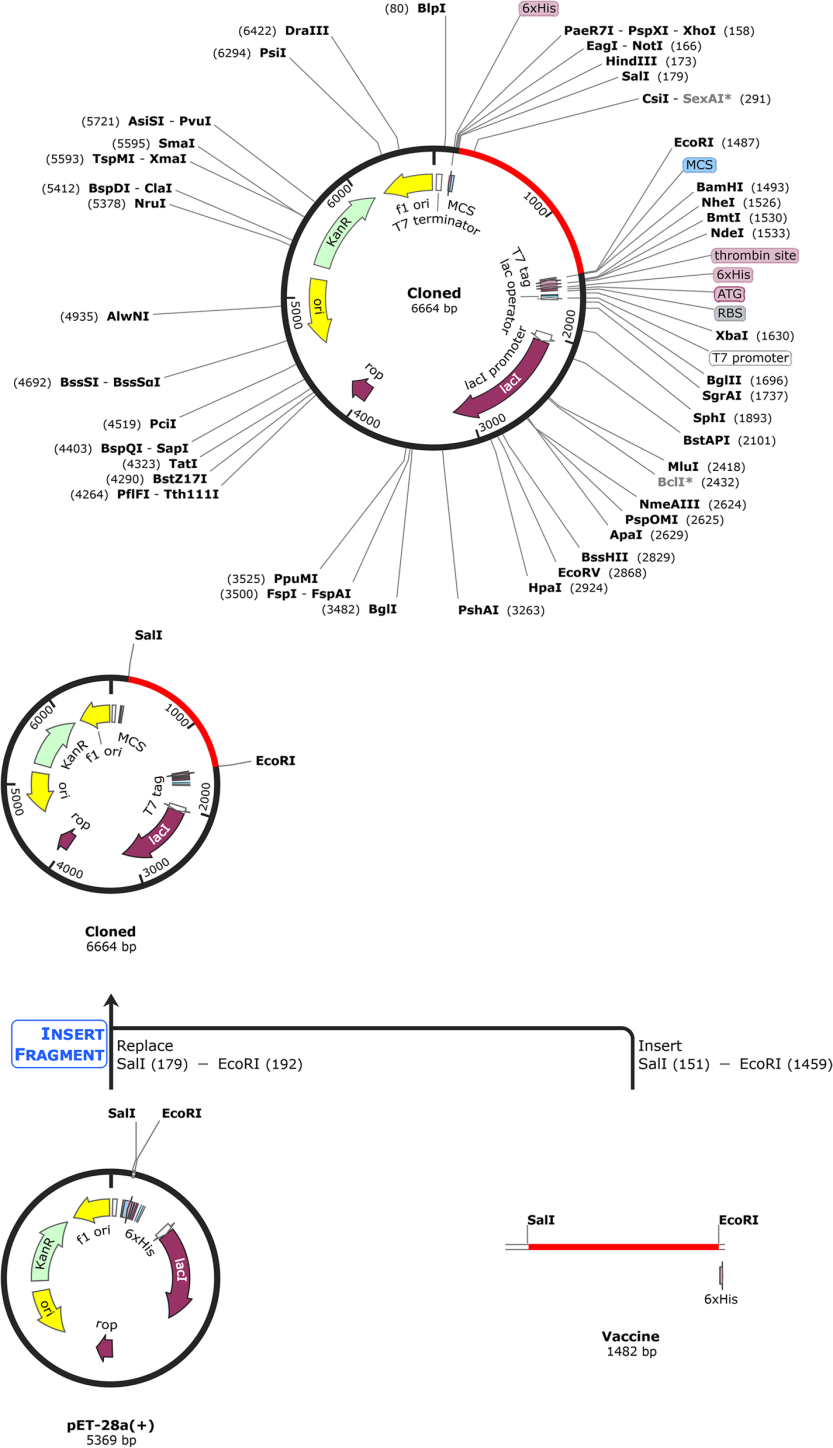
Improved and optimized vaccine was inserted into pET-28a (+) *E. coli* expression vector using SnapGene 4.2 software for *in-silico* cloning. The red color indicates the gene of interest. The black color represents the expression vector pET-28a (+)

The JCat tool optimized the vaccine sequence for efficient expression in *E. coli* bacteria with a 52.72% GC content and a CAI value of 0.996. The SalI and EcoRI restriction sites were utilized to insert the optimized DNA sequence into the *E. coli* vector PET28a( +). A 6.6 kbp clone (Fig. 10) was constructed, and the recombinant vaccine was purified with immune chromatography using a 6-histidine tag.

### mRNA secondary and tertiary structure prediction

**Fig. 11:**
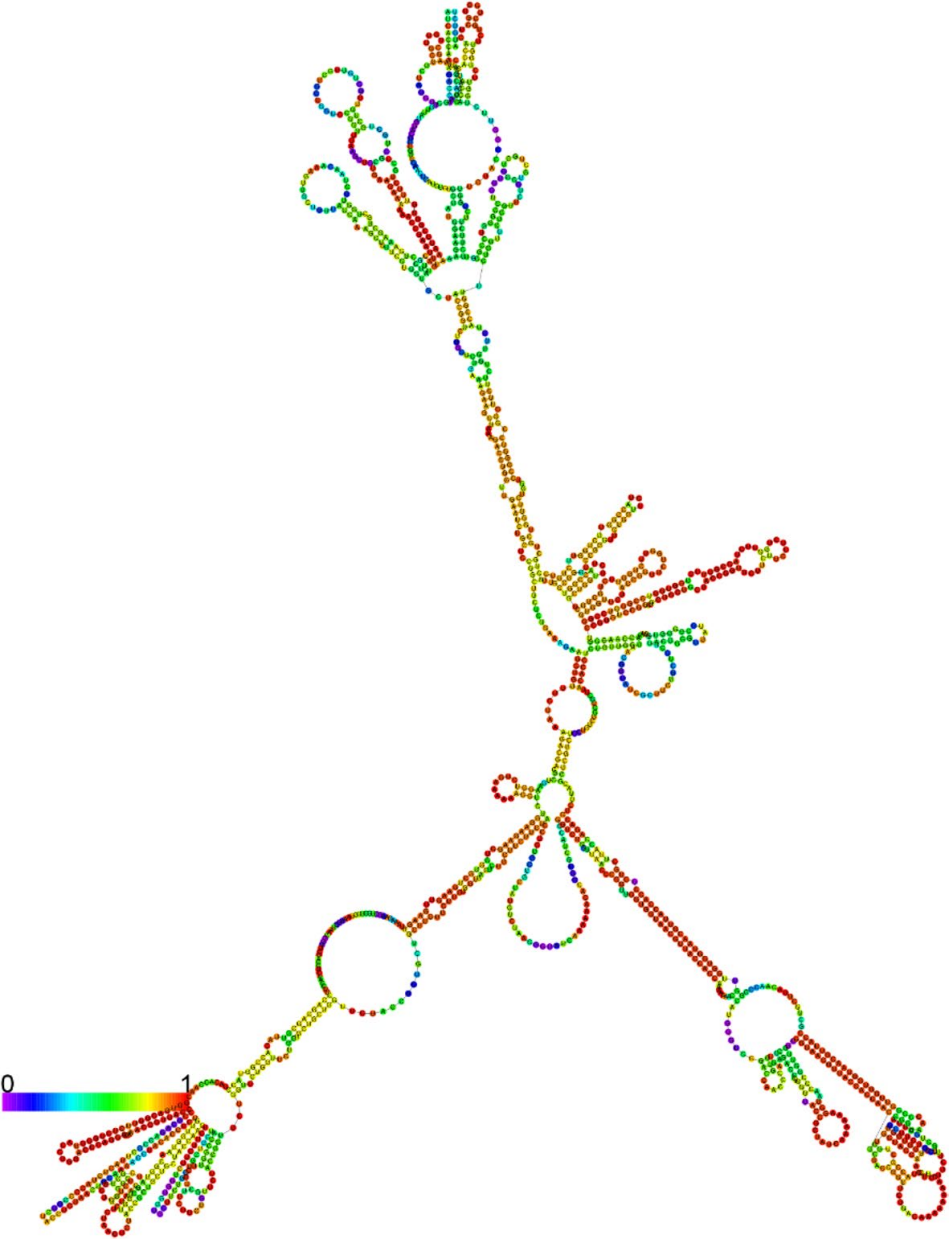
Predicted centroid secondary structure of mRNA of the vaccine construct

**Fig. 12:**
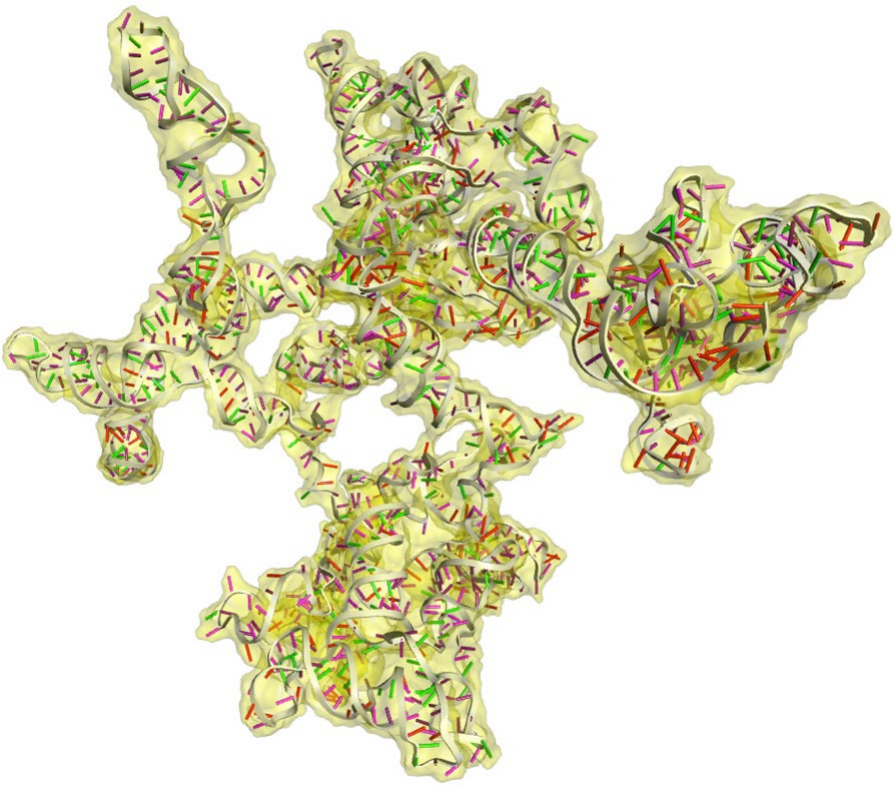
Tertiary structure of mRNA of the vaccine construct

The optimized DNA sequence of the vaccine was converted into an RNA sequence to construct the mRNA vaccine. RNAfold was used to generate the mRNA’s secondary structure, which had minimal free energy of − 452.10 kcal/mol, as depicted in Fig. 11. The 3D structure of mRNA is shown in Fig. 12.

### Immune simulation analysis

**Fig. 13:**
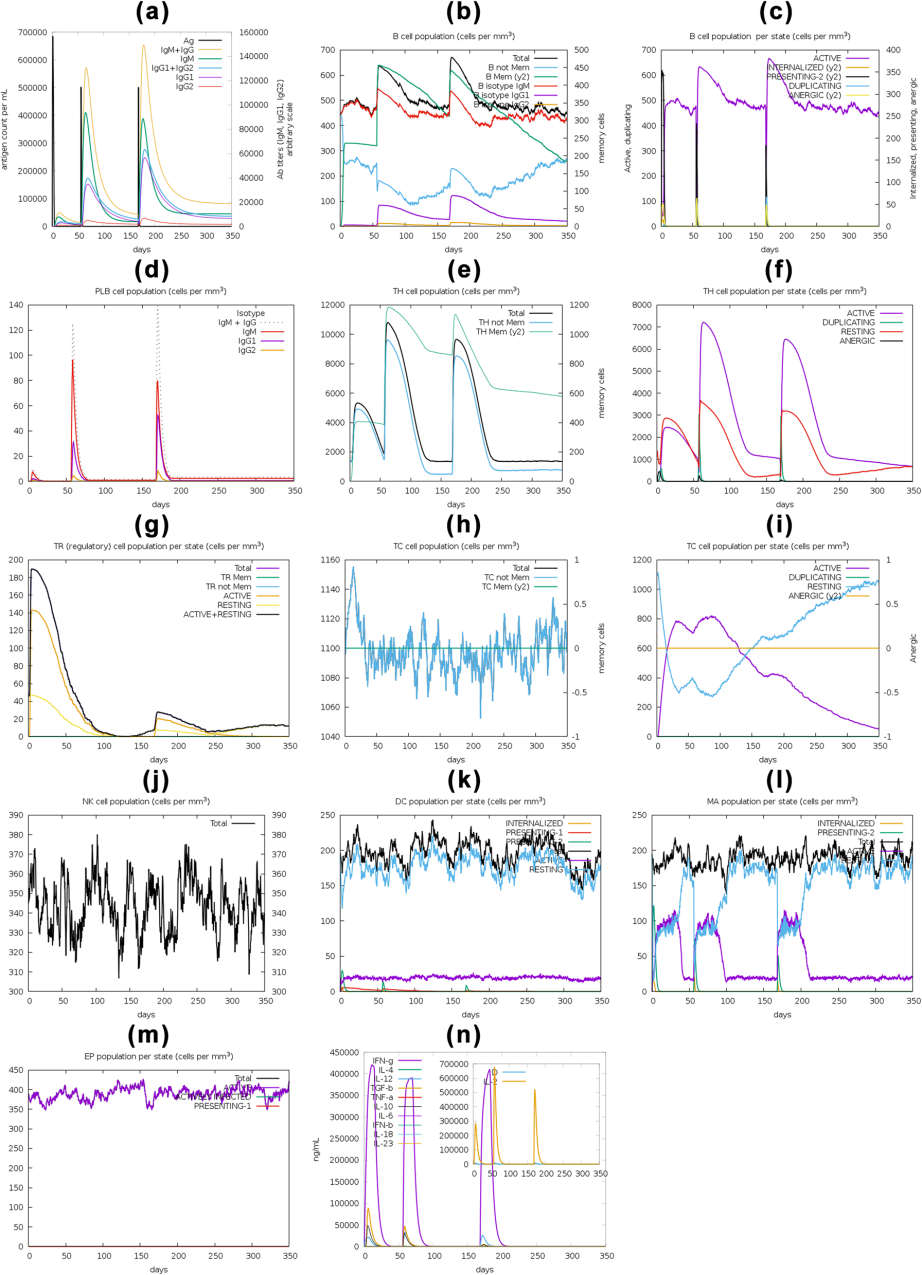
An in silico immune response simulation was performed on the designed vaccine with 3 shots for 350 days. Various parameters were assessed, including antigen and immunoglobulins (**a**), B cell (**b**–**d**), T cell (**e**–**i**), natural killer cell (**j**), dendritic cell (**k**), macrophage (**l**), epithelial presenting cell (**m**) populations, and cytokine concentration (**n**). Simpson index (D) was used to evaluate the simulation results

C-ImmSim reported that the primary immune response was significantly stimulated by the gradual increase in immunoglobulin levels such as IgG, IgG1, IgG2, and IgM after each of the three vaccine doses. However, the concentration of immunoglobulins was highest immediately after the vaccine was administered and decreased over time. The concentration of immunoglobulins was significantly higher after the third dose. In contrast, the antigen concentration decreased during and after the vaccine’s second and third doses, as illustrated in Fig. 13a. The active and total B cell populations remained elevated, as shown in Fig. 13b, c. The concentration of plasma B cells increased for several days after the vaccination (Fig. 13d). The active and total helper T cells were elevated and sustained after administering the vaccine (Fig. 13e, f). The active and resting helper regulatory T cell concentrations were highest after the first shot of the vaccine and gradually decreased over time (Fig. 13g). The concentration of cytotoxic helper T cells varied over time (Fig. 13h). Their active form decreased with constant energy after vaccination doses (Fig. 13i). The population of natural killer cells also fluctuated during the vaccination process (Fig. 13j). The concentrations of dendritic cells, macrophages, and epithelial presenting cells were evaluated in cells per mm^3^, as shown in Figs. 13k–m. The activation of different cells resulted in the elevation of different cytokine and interleukin concentrations after the vaccine (Fig. 13n).

## Discussion

Developing vaccines against fungi is challenging due to their ability to change shape [82]. Fungal infections typically begin with inhaling spores, which the immune system is generally effective at killing. However, if the immune system fails, an infection can occur. Unfortunately, vaccines for invasive mycoses and other fungal infections are currently unavailable, and antifungal drugs are limited, resulting in a poor prognosis for these diseases. *C. neoformans* infections can be severe for immunocompromised individuals, but vaccines for fungal diseases like cryptococcosis are still in development. Several potential vaccine types for cryptococcal meningitis exist, including whole organism vaccines, subunit recombinant protein vaccines, and mRNA vaccines.

In recent years, there has been a shift towards using multi-omics approaches for vaccine development, which utilize bioinformatics and structural biology tools to generate epitope-based vaccines. These studies, which focus on the antigenic parts of the pathogenic microorganisms, have shown promising results and represent a significant percentage of vaccine development research [83, 84]. However, developing an effective vaccine against *C. neoformans* has been challenging due to the fungus’s complex nature, genetic plasticity, and lack of broadly applicable testing. The current study employed a reverse vaccinology approach to generate a shortlist of potential vaccine candidates based on analyzing the MPs of *C. neoformans* through immunoinformatic computational tools. The objective was to identify conserved vaccine candidates that provide coverage against various pathotypes before proceeding to the subsequent stage of wet lab validation. A multi-epitope vaccine was designed based on the filtered vaccine candidates [85].

Using the immunoinformatics approach, the outer cell wall protein (MP) of *C. neoformans* was selected as the target for vaccine design, following successful application against various pathogens. A recent study proposed a multitype vaccine against COVID-19 using a deep learning approach for prediction and design. This study utilized a similar methodology to prior successful vaccine designs, emphasizing developing a potential vaccine that provides coverage against the majority of *C. neoformans* pathotypes and investigating its characteristics rather than introducing novel prediction techniques [86–88].

The present study utilized online web servers to specify vaccine candidates against *C. neoformans* pathotypes that were highly conserved. The outer cell wall protein, MP, was selected due to its high antigenicity score (0.876) and non-homology to human proteins for safety in clinical trials. MP is essential for fungal shape maintenance and survival. Fungal MP cell wall heavily glycosylated protein plays a vital role in fungal physiology and pathogenesis, such as cell–cell recognition, cell surface protection, and interaction with the host immune system [35, 89]. Additionally, their accessibility to the host immune system makes them promising candidates for vaccine development [90]. MPs in the fungal cell wall are promising targets for drugs and vaccines against fungal infections. Enzyme preparations containing protease and β-glucanase have been authorized for extracting MPs from yeast walls. However, the development of drugs targeting β-1,3-glucan synthesis has been more successful than vaccines targeting MPs. Vaccine development against the MPs is a promising target to stop the growth of fungi accompanied by drugs.

Utilizing epitopes that have been mapped for constructing a vaccine is an advanced approach to eliciting an immune response against infectious agents [91]. However, relying solely on peptide vaccines has its limitations. Single peptide epitopes may not be potent enough to trigger a robust and sustained immune response, as they possess low immunogenicity and may be unstable, getting degraded by human proteolytic enzymes before inducing an immune response [92]. Consequently, the present study proposes a multi-epitope vaccine that combines peptides with suitable linkers, filtered through various criteria to select conserved, highly antigenic, immunogenic, cytokine-inducing, non-allergenic, and non-toxic epitopes. Multi-epitope vaccines are superior to monovalent ones as they stimulate efficient humoral and cellular immune responses [93].

In this study, different CTL binding epitopes having antigenic, immunogenic, non-toxic, and non-allergic were sorted after predicting through the NetMHCpan EL4.1 method. Accordingly, five CLT epitopes were selected for the vaccine candidate against *C. neoformans* due to their high antigenic score, as shown in Table 2 and Fig. 3. Correspondingly, multiple HTL epitopes with antigenic, non-toxic, non-allergic, IFN-gamma, IL4, and IL10-inducing properties were identified. However, despite this, only five HTL binding epitopes were chosen because of higher antigenicity, as mentioned in Table 3 and Fig. 3. In the same way, five LBL epitopes with higher antigenicity, non-toxicity, and non-allergenicity were preferred for the *C. neoformans* vaccine candidate (Table 4 and Fig. 3).

The final vaccine construct included adjuvants like 50S ribosomal protein L7/L12 to generate to settle the antigenic components consistently released over time, improving the immune response and activating TLR. This outcome overcomes one of the main limitations of peptide vaccines [55, 94–96]. Effective linkers were utilized to connect preferred epitopes from each protein candidate, providing sufficient separation between them [97]. Initially, EAAAK was utilized to enhance the bi-functional catalytic activity and rigidity while also improving the stability of the fusion protein [98]. The AAY linker is used to connect CTL epitopes in a natural form and prevent the formation of junctional epitopes, improving multi-epitope vaccine presentation. GPGPG was chosen for its ability to induce HTL immune response and restore immunogenicity of individual epitopes by breaking junctional immunogenicity [99]. The KK linker was selected for its pH-regulating properties close to the physiological range [100].

Based on previously published studies [73, 74, 81, 101–103], a stable potential vaccine construct was developed consisting of 490 residues with a molecular weight of 45.58 kDa, a slightly acidic pI of 6.18, high antigenicity score of 1.4344, and high solubility score of 0.88. The vaccine was developed by incorporating selected LBL, HTL, and CTL epitopes, linkers, adjuvant, and 6 × His Tag. The vaccine demonstrated high population coverage across different world continents (Fig. 2), and its secondary and tertiary structures were predicted (Table 6 and Figs. 4 and 5). The vaccine was identified to be antigenic, as well as non-allergenic and non-toxic. The 3D structure of the vaccine was then validated by the Ramachandran plot (Fig. 6), ERRAT, and VERIFY3D. Further, five linear and fourteen discontinuous B cell epitopes (Tables S3 and S4 from supplementary material and Fig. 7) confirmed the ability of the conjugate vaccine to activate the B cells for antibody production.

After the inoculation of the vaccine, its primary goal is to activate the immune response against the foreign antigen. For this purpose, TLRs recognize and respond to molecules from pathogens to activate innate immunity. Several TLRs are involved in fungal recognition, but the most important ones are TLR2 and TLR4. TLR2 can form complexes with TLR1 or TLR6 to detect various fungal cell wall components, such as mannoprotein. However, TLR4 can recognize fungal mannans and β-glucans. In addition, TLR2 and TLR4 can cooperate with other receptors, such as Dectin-1, to enhance the immune response to fungi. TLRs can activate macrophages, neutrophils, and dendritic cells to produce inflammatory cytokines and kill fungi^135^. They can also polarize adaptive immunity by inducing Th1 or Th17 responses^146^. For this purpose, the vaccine docked with TLR2, TLR4, and TLR6 receptors on different immune cells’ surfaces. The docking results of the vaccine with TLR2, TLR4, and TLR6 confirmed significant − 1413.7, − 1413.7, and − 1390.2 kcal/mol of binding energies of the complexes, respectively (Table 7 and Fig. 8). In addition, the molecular simulation results showed the mobility, deformability, B-factor, eigenvalues, variance, and covariance of the vaccine with TLR2, TLR4, and TLR6 complexes (Fig. 9).

The expression of the vaccine construct was then analyzed by in silico cloning, as shown in Fig. 10. Further, the finalized DNA sequence was transcribed into mRNA. Then, their secondary (Fig. 11) and tertiary (Fig. 12) structures were predicted. Finally, the in silico immune response of the conjugate vaccine was validated by inoculating three vaccine shots of 1000 antigens with eight and then 24 weeks of intervals after the 1st shot for a total of 350 days. The production of all the required immune cells, interferons, and other pro-inflammatory cytokines against the vaccine was produced with different concentrations at different times after the inoculation of the vaccine, as shown in Fig. 13.

Currently, few efforts have been made to suggest vaccines against fungi, and the development of such vaccines has not been successful. The futile experiments stem from the fungal’s subtle differences in pathogenesis, host–pathogen interactions, and immune responses. Hence, the study has utilized a systemic immunoinformatic approach to develop a potent multi-epitope-based fungal vaccine. However, despite the potential of the immunoinformatic approach, there may be limitations due to the absence of a standard benchmark for vaccine development against fungi and limited knowledge of their pathogenesis and adaptive immune system response. Consequently, to evaluate the immunogenicity, efficacy, and safety of the newly developed vaccine, experimental validation is required both in vivo and in vitro.

## Conclusion

In silico vaccine design utilizing computational approaches was performed to identify a potential candidate for clinical trials. The study constructed an effective vaccine against MP of the *C. neoformans* to achieve good population coverage and immune response. By employing immuno-informatics techniques, T and B cell multi-epitope vaccines were designed. Molecular docking was conducted with ClusPro, demonstrating binding energies of − 1413.7, − 1413.7, and − 1390.2 kcal/mol with TLR2, TLR4, and TLR6, respectively, and the Ramachandran plot indicating a favored region of 93.7%. The vaccine construct was found to have good protein expression as determined by the SnapGene tool. Moreover, in silico trials demonstrated a strong immune response to the vaccine against MP of the *C. neoformans*. The proposed vaccine construct fulfilled the criteria for antigenicity, immunogenicity, allergenicity, toxicity, and other physicochemical properties, suggesting it is stable and safe. However, preclinical studies and authentication are required before experimental clinical trials can be conducted to confirm the study results.

### Supplementary Information

**Additional file 1: Table S1.** NetMHCpan EL 4.1 method on IEDB server predicted antigenic CTL binding epitopes with their alleles of MP in C. neoformans. **Table S2.** MP of C. neoformans antigenic HTL binding epitopes with their alleles predicted using IEDB recommended 2.22 method on the IEDB server. **Table S3.** Predicted Linear Epitopes of vaccine of MP of C. neoformans. **Table S4.** Predicted Discontinuous Epitopes of vaccine of MP of C. neoformans.

## Data Availability

The corresponding author [AE] can be contacted to obtain the available data which support the findings of this study.

## Abbreviations

HIV/AIDS: Human immunodeficiency virus/acquired immunodeficiency syndrome
CDC: Centers for Disease Control and Prevention
CNS: Central nervous system
MPs: Mannoproteins
HTL: Helper T lymphocyte
CTL: Cytotoxic T lymphocyte
IFN: Interferons
IL: Interleukins
TLR: Toll-like receptors
PDB: Protein Data Bank
